# Copper inhibits protein maturation in the secretory pathway by targeting the Sec61 translocon in *Saccharomyces cerevisiae*

**DOI:** 10.1016/j.jbc.2022.102170

**Published:** 2022-06-20

**Authors:** Nitu Saha, Raghuvir Singh Tomar

**Affiliations:** Laboratory of Chromatin Biology, Department of Biological Sciences, Indian Institute of Science Education and Research, Bhopal, Madhya Pradesh, India

**Keywords:** copper, endoplasmic reticulum, protein translocation, cell biology, protein processing, BCS, bathocuproinedisulfonic acid disodium salt, CPY, carboxypeptidase Y, ER, endoplasmic reticulum, ETA, ethanolamine, *EV*, empty vector, Gas1, glycophospholipid-anchored surface protein, GEF, guanine nucleotide exchange factor, GPI, glycosylphosphatidylinositol, ROS, reactive oxygen species, SC, synthetic complete, TCA, trichloroacetic acid, Tm, tunicamycin, TM, transmembrane, UT, untreated, VSG, variant surface glycoprotein

## Abstract

In *Saccharomyces cerevisiae*, proteins destined for secretion utilize the post-translational translocon machinery to gain entry into the endoplasmic reticulum. These proteins then mature by undergoing a number of post-translational modifications in different compartments of the secretory pathway. While these modifications have been well established for many proteins, to date only a few studies have been conducted regarding the conditions and factors affecting maturation of these proteins before entering into the endoplasmic reticulum. Here, using immunoblotting, microscopy, and spot test assays, we show that excess copper inhibits the Sec61 translocon function and causes accumulation of two well-known post-translationally translocated proteins, Gas1 (glycophospholipid-anchored surface protein) and CPY (carboxypeptidase Y), in the cytosol. We further show that the copper-sensitive phenotype of *sec61*-deficient yeast cells is ameliorated by restoring the levels of *SEC61* through plasmid transformation. Furthermore, screening of translocation-defective Sec61 mutants revealed that *sec61-22*, bearing L80M, V134I, M248V, and L342S mutations, is resistant to copper, suggesting that copper might be inflicting toxicity through one of these residues. In conclusion, these findings imply that copper-mediated accumulation of post-translationally translocated proteins is due to the inhibition of Sec61.

Copper, an essential element, is required by multiple enzymes, such as lysyl oxidase, cytochrome C, superoxide dismutase, and others (1). Copper sensing in yeast and ergo, the decision for its uptake, or sequestration/export are mediated primarily by two transcription factors, Ace1 and Mac1 (2). If the cells sense excessive copper in the extracellular milieu, then Ace1 undergoes a change in its conformation to convert into an active form. This activated transcription factor binds to the promoter of genes like *CUP1*, *CRS5* (metallothioneins), as well as *SOD1*, which serves as a reservoir of ligand-bound copper in the intracellular milieu as the excess copper may induce production of free radicals resulting in cellular destruction (3). On the contrary, in the copper-deficit condition, the copper uptake genes are activated by Mac1 (4). Since the mammalian system inhabits a number of orthologs of copper metabolism–related enzymes in yeast, in-depth studies in this simple eukaryote have provided critical insights into the mammalian system. The mammalian orthologs of copper metabolism are stringently regulated by co-ordination of multiple transcription factors, which reflect maintenance of this essential metal in extremely different and unique levels from cells to organs (5). As a result, any perturbation in copper metabolism results in serious consequences. For example, Menkes disease because of mutations in the *ATP7A* gene is characterized by intestinal copper accumulation, whereas its depletion in the enzymes of the peripheral region (6, 7) caused Wilson’s disease, because of mutations in the *ATP7B* gene, which is characterized by excessive copper accumulation in the neuronal and hepatic tissues (8, 9, 10). Thus, copper homeostasis is maintained through tight coordination of uptake, transport, and excretion (11).

Sec61 complex is a heterotrimeric channel for protein conduction used by both cotranslational and post-translational translocation pathways (12). In yeast, the translocon is composed of three proteins, Sbh1, Sec61, and Sss1 (13, 14). The core structure of the Sec61p, approximately 53 kDa protein, comprises 10 transmembrane (TM) helices. These helices are separated by loops, out of which L6 plays a role in translocation of proteins, whereas L8 contains a highly conserved residue, which is positively charged and provides a major ribosome-binding site (15). Sbh1, the beta subunit of translocon, is a small protein of approximately 8.7 kDa. Structurally, it has a single TM domain at C-terminal region and a cytosolic N-terminal region previously thought to have guanine nucleotide exchange factor (GEF) activity. However, it is no longer believed to contain a GEF activity. Both Sbh1 and its homolog Sbh2 are nonessential yeast genes and that the translocation defects of a Δ*sbh1*Δ*sbh2* mutant can be suppressed by expression of the TM span of Sbh1, which argues against the presence of a cytoplasmic GEF domain (16, 17, 18). Sss1 is an essential small protein of the complex, which is an integral membrane protein with its amino terminal exposed to the cytosol (19). Multiple structural studies of the trimeric complex from different species have revealed details about the mechanism through which the polypeptides enter the protein-conducting channel to reach into the lumen or become associated with the endoplasmic reticulum (ER) membrane. X-ray structures of protein-conducting channel from *Methanococcus jannaschii* (14) and monomeric yeast Sec61 complexes (20) have revealed the plausible structural changes in the translocation when shifting from idle (not engaged by polypeptide) state to active state (engaged by polypeptide). In its idle state, the channel pore remains closed by a plug domain and an additional ring of hydrophobic residues. Together, they block the outer cytoplasmic environment from the inner ER lumen. However, when the signal sequence bearing polypeptide approaches the channel, the interactions holding the plug in its position are destabilized, and so the plug shifts making room for the polypeptide to pass through. The ring of hydrophobic residues forms a seal around the passing polypeptide to prevent the entry of small molecules from the cytoplasm into the lumen (14, 20). In the case of cotranslational translocation, only the association between protein-conducting Sec61 and the ribosome’s exit tunnel is required (20, 21). While for post-translational translocation, the complex partners with Sec62–Sec63 complex. The Sec62–Sec63 complex is composed of two essential subunits, Sec62p and Sec63p, and two nonessential subunits, Sec71p and Sec72p (22, 23).

Plethora of proteins, utilizing the secretory pathway, is synthesized in the cytosol in their inactive form. These proteins are translocated to ER, where they are folded and post-translationally modified and then transferred to Golgi, where further modifications take place to finally reach their diverse destinations in the active form (24). For example, glycophospholipid-anchored surface protein (Gas1), a β-(1, 3)-glucan elongase protein, is post-translationally modified through the attachment of a glycolipid moiety called glycosylphosphatidylinositol (GPI) at their C-terminal end. This attachment facilitates the anchoring of protein to the plasma membrane’s outer leaflet of the lipid bilayer (25, 26). Similarly, carboxypeptidase Y (CPY) is synthesized in the form of preproenzyme. The preproenzyme enters in the ER lumen through post-translational translocation, where the signal sequence is cleaved resulting in proCPY after which the enzyme gets folded through disulphide bond formation and glycosylation to give the p1CPY form; the enzyme is then transported to Golgi, where the outer mannose residues are added giving rise to p2CPY. Finally, inside the vacuole, proteinase B cleaves the p2CPY to form the mature CPY (27).

Molecules specifically inhibiting the translocon channel may aid in elucidating the underlying functional mechanisms of the complex. A number of molecules from different sources have shown the potential of inhibiting the translocation of cotranslationally and post-translationally targeting proteins. In case of apratoxin A, the molecule has been shown to hamper the cotranslational translocation in *in vitro* condition (28), whereas group of molecules called cotransins inhibits specific translocating substrates by targeting the Sec61α (29, 30, 31).

More recently, a new natural metabolite isolated from the organism *Chaetosphaeria tulasneorum* has been shown to target the Sec61 translocon machinery of the yeast and mammals. The translocation of both cotranslationally and post-translationally translocating substrates was inhibited. Furthermore, dominant mutations of Sec61, isolated, post the mutagenesis and genome sequencing of yeast and mammalian cells, conferred resistance against the isolated metabolite. Finally, these resistant mutants were shown to exhibit the *prl* phenotype. *prl* mutants mimic partially opened channel of Sec61 and so allow the passing of defective signal bearing secretory proteins, which are otherwise blocked by the WT Sec61. Thus, it was concluded that the metabolite most probably binds to the closed translocon structure so that it fails to open for the efficient translocation of proteins (32).

Reports suggest that copper effects the glycosylation levels of secretory proteins in multiple cell lines (33). The underlying reason for the phenomenon is still obscure. While the role of transition metals in reactive oxygen species (ROS) generation or in the replacement of metals in metallothioneins is extensively studied, its effect on the maturation of secretory proteins has never been explored (34, 35, 36). In an attempt to explore the possible role of these metals in the maturation of secretory proteins, we tested the effect of multiple metals on the secretory protein maturation (Gas1, data not shown) and found that treatment with copper leads to accumulation of immature form. In this communication, we attempt to address the question by checking the effect of copper on two different secretory proteins, Gas1 and CPY. We first report the selective accumulation of immature Gas1 by copper (CuCl_2_.2H_2_O) treatment of yeast cells. The accumulation of immature Gas1 protein was reversed by treatment of cells with ethanolamine (ETA) and specific copper chelator bathocuproinedisulfonic acid disodium salt (BCS). The molecular weight of the immature protein revealed that the Gas1 protein was throttled as unglycosylated form. Tunicamycin (Tm) treatment in WT yeast cells has been shown to accumulate unglycosylated form of red fluorescent protein-Gas1 (37). We found that the form of copper accumulated showed similar migration as the Tm-treated Gas1. Furthermore, we found that another signal recognition particle–independent protein CPY, in the presence of copper, accumulated as a slower migrating protein band than the Tm-treated bands pointing at the accumulation of proproteins (38, 39). We reasoned, if copper treatment leads to accumulation of protein in the prepro form, then the compound must be targeting the translocon. Both Gas1p and CPY utilize the Sec61 complex to gain access into the ER lumen where further post-translational modifications occur (26, 38). On testing various components of the translocon complex, cells with lower levels of Sec61p showed compromised growth in the presence of copper. The growth-sensitive phenotype of Sec61 mutant in copper is rescued upon restoration of Sec61 levels through plasmid. The *sec61-DAmP* yeast cells reported to accumulate precursor Gas1p (40) also accumulated the preproCPY. The immature protein band of CPY corresponds to the band accumulated under copper treatment. We also tested Sec61 translocation-defective mutants (27) in copper and found that only *sec61-22*, inhabiting mutations at L80M, V134I, M248V, and L342S, was resistant to copper. Thus, our results strongly suggest that copper may inhibit the Sec61 translocon–mediated entry/release of these proteins into the ER lumen.

## Results

### Copper causes accumulation of precursor Gas1p in dose- and time-dependent manner

**Figure 1:**
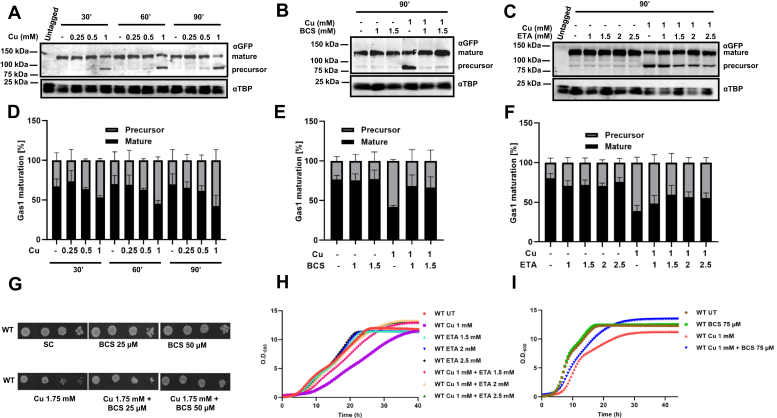
**Copper causes accumulation of precursor Gas1p in dose- and time-dependent manner.***A*, immunoblots of Gas1-GFP–transformed BY4743 cells treated with increasing concentrations of copper CuCl_2_.2H_2_O (0.25 mM, 0.5, and 1 mM). Transformed cells were grown till an absorbance ∼1 at 600 nm and then left untreated (−) or treated with indicated concentrations of copper, and samples were collected at 30′, 60′, and 90’. Proteins extracted from these samples were subjected to Western blotting (described in the Experimental procedures section). *B*, Western blot of the same cells subjected to BCS (1 and 1.5 mM) or copper (1 mM) or both treatments and harvested at 90’. *C*, ETA rescue of the same cells treated with either increasing concentrations of ETA (1, 1.5, 2, 2.5 mM, respectively) or copper (1 mM) or both. TBP levels served as control. *D*–*F*, quantification of the Western blots from (n = 3) independent biological repeats (described in the Experimental procedures section). Graphs plotted as mean ± SD. *G*, spot test assay of BY4743 (WT) cells in plates containing either BCS (50, 75 μM), copper CuCl_2_.2H_2_O (Cu 1.75 mM), or both. Spots were grown for 3 days and photographed. *H*, growth curves of BY4743 (WT) cells either in the presence of copper (1 mM) or ETA (1.5, 2, and 2.5 mM) or both. *I*, growth curves of BY4743 (WT) cells either in the presence of copper (1 mM) or BCS (75 μM) or both. Graphs represent the absorbance at 600 nm values taken every 30′ for the indicated hours. BCS, bathocuproinedisulfonic acid disodium salt; ETA, ethanolamine; Gas1, glycophospholipid-anchored surface protein; TBP, TATA-binding protein.

**Figure 2:**
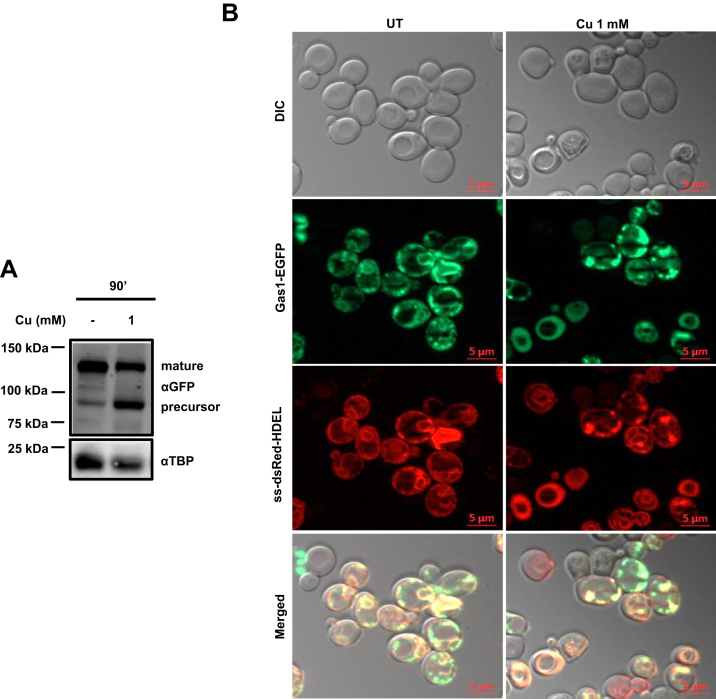
**Immature Gas1p accumulated under copper appears to be in the cytosol.***A*, immunoblots of Gas1-GFP–transformed GSHY583 cells treated with copper CuCl_2_.2H_2_O (1 mM). Transformed cells were grown till an absorbance reached ∼1 at 600 nm and then left untreated (−) or treated with indicated concentration of copper. Samples were collected at 90’. Proteins extracted from these samples were subjected to Western blotting (described in the Experimental procedures section). TBP served as control. *B*, microscopy images of Gas1-GFP–transformed GSHY583 cells, which were grown till an absorbance of ∼1 at 600 nm and then left untreated (UT) or treated with indicated concentration of copper for 90’. Bar represents 5 μm. ER, endoplasmic reticulum; Gas1, glycophospholipid-anchored surface protein; TBP, TATA-binding protein.

To investigate the effect of copper on Gas1p maturation, BY4743 yeast cells transformed with pRS415 Gas1-GFP (41) were treated with different copper concentrations, *viz.* 0.25, 0.5, and 1 mM, and cells were harvested at different time points, *viz.* 30′, 60′, and 90’. Following the harvest, we extracted the proteins and performed immunoblotting. Our result (Fig. 1*A*) clearly shows that the accumulation of precursor Gas1p is proportional to the concentration of copper used, and the time for which cells were exposed to copper (Fig. 1, *A* and *D*) shows that treatment with 1 mM copper at 90′ led to accumulation of the highest amount of Gas1p precursor form at 58% ± 14% compared with the untreated (UT) at 31% ± 13%. BCS is a specific copper chelator that is routinely used in yeast biology to reverse the effect of copper (42). To prove that copper is responsible for the accumulation of the immature protein, we cotreated cells with copper and BCS. The result (Fig. 1*B*) shows that indeed the addition of BCS rescues the accumulation of precursor form. While UT or copper-treated cells accumulated immature proteins at 24% ± 5% and 58% ± 2%, respectively; addition of BCS reduced the accumulation to about 32% ± 14% or 34% ± 14% at concentrations of 1 and 1.5 mM BCS, respectively (Fig. 1*E*). Furthermore, we checked another copper chelator, ETA, which is known to form complex with copper (43). Upon cosupplementation, the accumulation of immature form was reversed in a dose-dependent manner (Fig. 1, *C* and *F*). We found while the UT and copper-treated cells accumulate about 20% ± 6% and 61% ± 7% immature form, respectively. Cotreatment with ETA at 2.5 mM concentration reduced the accumulation to 45% ± 7%. Spot test assay of untransformed BY4743 cells showed that the deleterious effect on cell growth by copper was ameliorated by BCS (Fig. 1*G*). We did growth curve analysis of untransformed BY4743 cells and found that 1 mM copper was able to exert effect on the growth of cells in liquid culture, which was ameliorated by cotreatment of ETA and BCS, respectively (Fig. 1, *H* and *I*). We also checked the response of the transformed cells in the presence of copper, which inhibited the growth with increasing concentration (Fig. S1*A*). Since Gas1p was tagged with GFP, we next tested the localization of Gas1-GFP signal in the cell through microscopy. To this end, we transformed the GSHY583 strain containing the N-terminal ER signal sequence, dsRed, and an HDEL tag (44). After confirming that the transformed GSHY583 cells treated with copper showed accumulation of Gas1 precursor form (Fig. 2*A*), we performed microscopy to assess the localization of this precursor form. Figure 2*B* shows that the copper-treated cells exhibited marked difference in Gas1 distribution compared with the UT cells. In the UT cells, Gas1 is present at the nuclear rim and the cortical ER region in the mother cells but only in the cortical ER in the new buds. However, in the copper-treated cells, the cortical distribution of the protein is majorily distorted with almost no puncta or tubes. Moreover, the copper-treated cells show an increase in the diffused cytoplasmic Gas1-GFP signal compared with the UT cells. Interestingly, we found that the signal sequence–attached DsRed protein also exhibited similar pattern as the Gas1 protein (Fig. 2*B*) under copper treatment. Inspection of the signal sequence–attached dsRed-HDEL revealed that it is a Kar2 signal sequence. As Kar2 partly utilizes signal recognition particle-independent translocation like Gas1 (45), it shows defective distribution upon copper treatment. Thus, we conclude that copper treatment results in accumulation of immature Gas1p.

### Copper causes accumulation of precursor Gas1p and preproCPY

**Figure 3:**
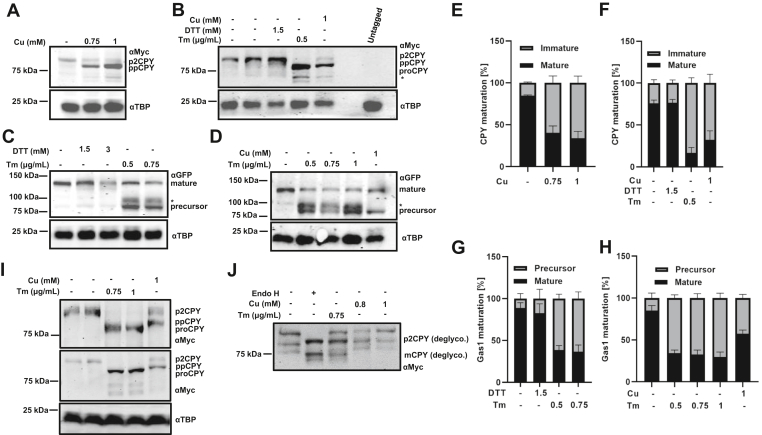
**Copper causes accumulation of precursor Gas1p and preproCPY.***A*, immunoblots of CPY-myc tagged BY4741 (WT) cells treated with increasing concentrations of copper. Transformed BY4741 cells were grown till an absorbance of ∼1 at 600 nm and then left untreated (−) or treated with indicated concentrations of copper (0.75 mM, 1 mM). Proteins extracted from samples collected at 90′ were subjected to Western blotting (described in the Experimental procedures section). *B*, immunoblots of the same cells untreated (lane 1) treated with DMSO (control for Tm, lane 2) or DTT (1.5 mM) or Tm (0.5 μg/ml) or copper (1 mM) and harvested at 90’. *C*, immunoblots of Gas1-GFP–transformed BY4743 cells untreated (−) or treated with DTT (1.5 and 3 mM) or Tm (0.5 and 0.75 μg/ml) for 90′ and harvested. *D*, immunoblot of Gas1-GFP–transformed BY4743 (WT) cells untreated (−) or treated with Tm (0.5, 0.75, and 1 μg/ml) or copper (1 mM) for 90′ and harvested. TBP served as control. *E*–*H*, quantification of the Western blots from (n = 3) independent biological repeats (quantification described in the Experimental procedures section). Graphs plotted as mean ± SD. *I*, immunoblots of CPY-myc tagged BY4741 cells treated with indicated concentrations of Tm or copper. Samples collected at 90′ were subjected to separation using 8 to 16% gradient gel (*top panel*) or 6% SDS gel (*lower panel*) and subjected to Western blot as described before. TBP served as control. *J*, immunoblots of CPY-myc tagged BY4741 cells treated with indicated concentrations of Tm or copper. Samples collected at 90′ were subjected to protein extraction. The lysate containing protein was incubated with Endo H or buffer. Postincubation, the samples were subjected to Western blot as before. (Note: Deglyco. stands for deglycosylated). CPY, carboxypeptidase Y; DMSO, dimethyl sulfoxide; Gas1, glycophospholipid-anchored surface protein; TBP, TATA-binding protein; Tm, tunicamycin.

Based on the existing studies, we hypothesized that the accumulation of immature Gas1p in the presence of copper might be the result of phosphatidylethanolamine pool depletion (46, 47) leading to a compromise in the addition of GPI anchor to Gas1 (47). To test this hypothesis, we checked the maturation of CPY, a non-GPI–anchored secretory protein. To this end, we endogenously myc tagged the CPY protein in BY4741 and tested the effect of copper on its growth (Fig. S1*B*). We then checked the effect of copper on CPY protein maturation. Results (Fig. 3, *A* and *E*) show the accumulation of immature CPY protein band under copper treatment, thereby ruling out the possibility that the absence of GPI anchor attachment might be causing the accumulation of immature Gas1p. The UT cells accumulated 15% ± 1%, whereas copper-treated cells accumulated 60% ± 8% and 66% ± 8% immature protein at concentrations of 0.75 and 1 mM, respectively. Slowly migrating bands of both, Gas1p and CPY, resembled the size of unglycosylated forms of these proteins. We debated, if the faster migrating band obtained under copper treatment is indeed the unglycosylated form, then it should correspond to the band accumulated under Tm treatment of these two proteins. Tm is a well-established inhibitor of N-linked glycosylation (48). Both CPY and Gas1p undergo N-linked glycosylation. Ergo, their unglycosylated form, accumulates under Tm treatment (27, 37). We treated cells with Tm at 0.5 μg/ml and observed that unglycosylated proCPY accumulated under Tm treatment, and the band migrates marginally faster than the copper-treated band of the protein (Fig. 3, *B* and *F*). Thus, the Tm-treated bands resemble the proCPY, and the copper-treated bands are preproCPY as reported in earlier studies (27). While the UT cells show 24% ± 4% of immature protein, Tm- (0.5 μg/ml) or copper (1 mM)-treated cells exhibit 83% ± 6% and 68% ± 11% levels of immature protein, respectively. In addition, we obtained a less prominent faster migrating band of CPY under Tm treatment (represented by asterisk [∗]) similar to the mature vacuolar form of nonglycosylated CPY consistent with the report that transport of CPY to the vacuole is delayed in Tm-treated cells (49). Since Tm is also an ER stress inducer, we tested the effect of DTT, another ER stress–inducing agent to check its effect on the protein maturation. The results (Fig. 3, *B* and *F*) show that DTT had no effect on the maturation of the CPY with immature band accumulation at 23% ± 4% compared with UT at 24% ± 4%. However, treatment of cells with higher concentration of DTT (5 mM) has been shown to accumulate the proCPY form (50); since we have used lower dose of DTT (1.5 mM), no accumulation of proCPY was observed. We next tested the fate of Gas1p under similar conditions. Our results (Fig. 3, *C* and *G*) clearly show that for both UT and DTT-treated (1.5 mM) samples, the immature protein levels were at 11% ± 6% and 17% ± 11%, respectively, whereas for 0.5 μg/ml, 0.75 μg/ml Tm-treated samples at 61% ± 5% and 63% ± 8%, respectively. Treatment of Tm (0.5, 0.75, and 1 μg/ml) or copper (1 mM) resulted in accumulation of immature Gas1p at 66% ± 4%, 67% ± 5%, 70% ± 6%, or 43% ± 4%, respectively, compared with 15% ± 6% under UT condition (Fig. 3, *D* and *H*). In addition, we repeatedly obtained an extra band in case of Tm treatment (represented by an asterisk ∗), which shows migration similar to the GPI-anchored form of ER Gas1p digested with EndoH (51). As can be seen in Figure 3*B*, copper treatment accumulates CPY band, which moves marginally slower than the proCPY accumulated under Tm treatment (38). We further resolved the bands using 8 to 16% SDS gradient gel or 6% SDS gel. Figure 3*I* clearly shows that in both gradient gel (*top panel*) as well as 6% gel (*bottom panel*), the copper-treated CPY band migrates slower than the Tm-treated proCPY consistent with the preproCPY form observed in case of translocation-defective *sec* mutants (38). To further confirm that the band seen under copper or Tm treatment represents the unglycosylated form, we performed EndoH assay. Figure 3*J* shows that the EndoH treatment of CPY in UT condition accumulates the deglycosylated (deglyco.) band, which migrates similar to the band obtained under copper or Tm treatment, thereby corroborating the fact that the bands indeed are the unglycosylated bands (52). Thus, our results strongly suggest that in the presence of copper, there is accumulation of immature form of Gas1p and CPY, which is independent of failure in GPI anchor attachment. Moreover, such immature proteins represent the precursor form of Gas1p and preproCPY.

Here, it is important to note that simple immunoblot approach employed to assess the maturation of Gas1p or CPY is less accurate as the method does not distinguish the proteins made before the addition of the perturbant (Cu, Tm, and DTT used in our case). Thus, commonly, the pulse chase labeling method is used to investigate the defects in yeast protein translocation. Therefore, it is possible that the presence of pre-existing protein before the addition of perturbant might be causing an underestimation of impact of copper treatment on Gas1 or CPY.

### *sec61-DAmP* strain is sensitive to copper treatment

**Figure 4:**
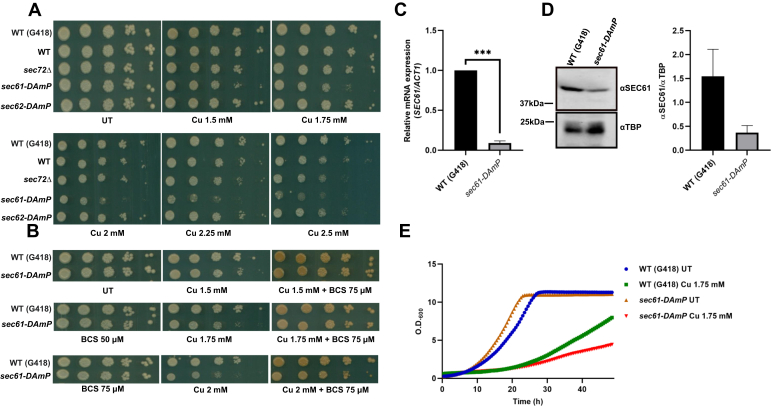
***sec61-DAmP* strain is sensitive to copper treatment.***A*, spot test assay of WT BY4741 with G418 in HO locus (WT [G418]), WT BY4741 without G418 in HO locus (WT), sec72*Δ, sec61-DAmP*, and *sec62-DAmP* cells (at 10-fold dilution, *left* to *right*) to test sensitivity in plates containing increasing concentration of copper CuCl_2_.2H_2_O (1.5, 1.75, 2, 2.25, and 2.5 mM). Untreated (UT) plate used as control shows uninhibited growth of WT and mutants. Cells were grown at 30 °C, and plates were scanned on fourth day. Representative images from three consistent independent repeats are shown. *B*, spot test assay showing the growth of (WT [G418]), *sec61-DAmP* in the presence of CuCl_2_.2H_2_O (1.5, 1.75, and 2 mM), BCS (50 μM, 75 μM) alone, or in combination. *C*, *SEC61* mRNA level was examined in WT (G418) and s*ec61-DAmP*, and the expression relative to *ACT1* is shown. Statistical analysis was performed using unpaired Student’s *t* test with Welch’s correction (in GraphPad Prism 8 GraphPad Software, Inc). Values shown are mean ± SD (n = 3). ∗∗∗*p* ≤ 0.001. *D*, Sec61 protein levels of indicated strains were determined through Western blot (described in the Experimental procedures section). TBP levels served as control. *E*, growth of the same cells was monitored in liquid media either in UT condition or in the presence of copper (1.5 and 1.75 mM). Graphs represent the absorbance at 600 nm values taken every 30′ for the indicated hours. BCS, bathocuproinedisulfonic acid disodium salt; TBP, TATA-binding protein.

Both Gas1p and CPY proteins enter into the ER lumen and are then glycosylated and modified by different enzymes, which target them through Golgi to their destinations at membrane or in vacuole, respectively (27, 38, 40). Under copper treatment, Gas1 accumulated as precursor form, which comigrated with the unglycosylated form obtained in Tm treatment. Microscopic examination of copper-treated cells showed that the protein exhibits marked difference in distribution compared with UT condition. Copper treatment causes accumulation of CPY proteins in the prepro form indicating that the signal sequence cleavage must not have happened. Together, the results indicate that there is accumulation of untranslocated version of protein with intact signal sequence (27, 40). Thus, we checked if translocation-defective mutants showed growth defect in the presence of copper. *DAmP* library of essential genes created by incorporation of kanamycin-resistance (Kan^R^) cassette in the 3′ UTR of the gene results in transcription of the gene under its natural promoter, but the resulting mRNA is unstable. Thus, the approach has proven useful in reducing the mRNA levels of essential genes by 4- to 10-fold (53, 54, 55). Many such essential genes of *DAmP* strains exhibit higher sensitivity in chemical agents than reported for their heterozygous counterparts (53). Therefore, we used the *DAmP* strains of *Saccharomyces cerevisiae* translocon genes to identify the target of copper. We checked the growth of translocation-defective cells on plates containing increasing copper concentrations and found that *sec61-DAmP* (but not *sec62-DAmP* or *sec72Δ*) was sensitive to copper (Fig. 4, *A* and *E*). This sensitivity was rescued by cotreatment of BCS (Fig. 4*B*). To corroborate our findings, we checked the levels of Sec61 w.r.t. WT (G418). The results (Fig. 4*C*) show that the levels of *SEC61* indeed are approximately fivefold lesser in the mutant compared with WT (G418). We further affirm that the observations are specific for *SEC61* as the levels of *SEC66* do not show any change between WT and the mutant (Fig. S2, *A* and *B*). *SEC61* level in UT *versus* copper-treated sample in WT (G418) showed a small but consistent difference (Fig. S2, *A* and *B*). We also found considerable difference in the levels of Sec61 protein in the WT (G418) *versus* the mutant strain (Fig. 4*D*). While the results suggest the possible role of Sec61 in mitigating copper toxicity, it is possible that the reduced translocation efficiency in the Sec61-deficient strain is synergistic with the defects of chaperone function or prosequence processing or errors in the protein processing, which may happen in the presence of copper. Together, these results suggest that the decreased level of Sec61 protein plays an important role toward copper sensitivity and that the protein might be targeted by copper to bring about the accumulation of immature form of Gas1 and CPY protein in yeast cells.

### *sec61-DAmP* sensitivity is ameliorated upon restoration of *SEC61* levels

If depleted levels of Sec61 protein are responsible for copper sensitivity, then the restoration of sec61 levels should ameliorate the sensitivity of cells. Thus, we transformed both WT (G418) and *sec61-DAmP* with the empty vector (*EV*) and *SEC61* expression plasmid and reconfirmed the identity of transformed cells using confirmatory primers (data not shown). Indeed, while the *EV* did not rescue the sensitivity of s*ec61-DAmP* strains in copper, the expression of *SEC61* through plasmid rescued the phenotype (Fig. S3*A*). Next, we checked the growth of these transformed cells in liquid media and found that the *SEC61* plasmid–transformed *sec61-DAmP* shows improved resistance to copper treatment (Fig. S3*B*). Furthermore, the levels of *SEC61* were restored in the *sec61-DAmP* strain transformed with *SEC61* expression plasmid but not in the *EV*-transformed strains (Fig. S3, *C* and *D*). *sec61-DAmP* has already been reported to accumulate the precursor form of Gas1p (40). We first showed that in the *EV*-transformed mutant, preproCPY is accumulated but not in the WT (G418) cells. Furthermore, *SEC61*-transformed mutant cells showed the restoration of CPY immature form. Thus, while the *EV* containing *sec61-DAmP* accumulates around 59% ± 6% of immature protein, the *SEC61* plasmid–transformed strain accumulates only 39% ± 4% (Fig. S3, *E* and *F*). Together, our results suggest that when the levels of *SEC61* are restored in *sec61-DAmP*, it overcomes the sensitivity toward copper by alleviating the translocation defect in the mutant.

### *sec61-DAmP* exhibits similar accumulation of precursor proteins as does copper-treated WT cells

**Figure 5:**
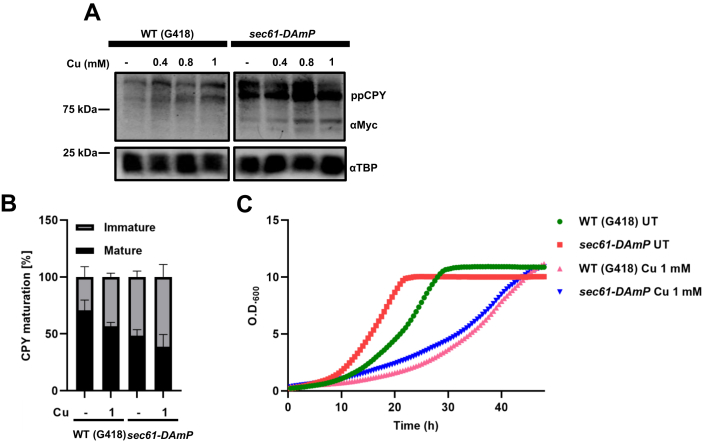
***sec61-DAmP* exhibits similar accumulation of precursor proteins as does copper-treated WT cells.***A*, immunoblots of myc-tagged CPY WT (G418), *sec61-DAmP* cells treated with increasing concentrations of copper (0.4, 0.8, and 1 mM) for 90’. Cells harvested were subjected to protein extraction and Western blot (described in the Experimental procedures section). TBP levels served as control. *B*, quantification of Western blots from (n = 3) independent biological repeats. Graphs plotted as mean ± SD. *C*, growth of the same cells was monitored in liquid media either in untreated (UT) condition or in the presence of copper (1 mM). Graphs represent the absorbance at 600 nm values taken every 30′ for the indicated hours. CPY, carboxypeptidase Y; TBP, TATA-binding protein.

Next, we sought to find out if the copper-accumulated protein comigrates with the protein accumulated in *sec61-DAmP* strains. To this end, we myc tagged the CPY protein in WT (G418) and *sec61-DAmP* strains. We treated the cells with indicated copper concentrations and harvested the cells at 90’. After the protein extraction from these cells, Western blot was performed. Copper treatment at 1 mM leads to accumulation of the protein band, which comigrated with the preproCPY in the *sec61-DAmP* strains (Fig. 5, *A* and *B*). We also checked the effect of copper (1 mM) on growth of the transformed strains (Fig. 5*C*). Thus, we conclude that copper treatment accumulates precursor form of CPY protein by targeting the Sec61 protein.

### Reduced Sec61 level is insensitive to either DTT or Tm; *sec61-22* is resistant to copper

**Figure 6:**
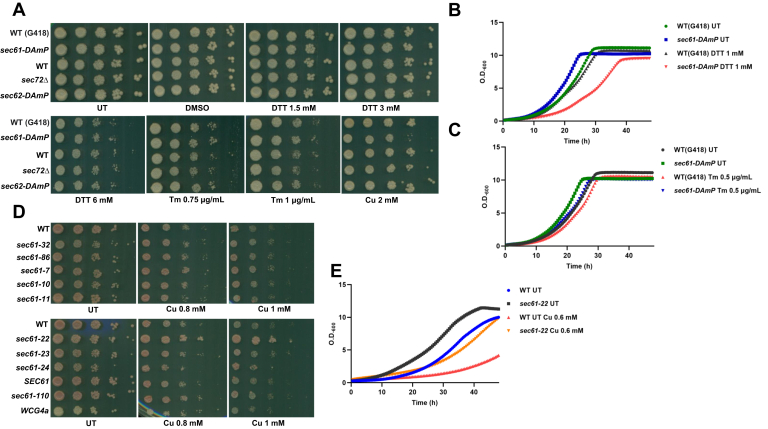
**Reduced Sec61 level is insensitive to either DTT or Tm; *sec61-22* is resistant to copper.***A*, same cells used for Figure 3 were spotted (at 10-fold dilution, *left to right*) in plates containing increasing concentration of DTT (1.5, 3, and 6 mM) or Tm (0.75 and 1 μg/ml) or copper (2 mM). Untreated (UT) plate and DMSO (for Tm) used as controls show uninhibited growth of WT and mutants. Cells were grown at 30 °C, and plates were scanned on fourth day. Representative images from three consistent independent repeats are shown. *B* and *C*, growth of the indicated strains was monitored in liquid media either in UT condition or in the presence of DTT (1 mM) (*B*) or Tm (0.5 μg/ml) (*C*). Graphs represent the absorbance at 600 nm values taken every 30′ for the indicated hours. *D*, spot test assay of the indicated strains in plates containing increasing concentration of copper CuCl_2_.2H_2_O (0.8 mm, 1 mm). UT plate used as control shows uninhibited growth of WT and mutants. Cells were grown at 30 °C, and plates were scanned on fourth day. Representative images from three consistent independent repeats are shown. *E*, growth of the WT and *sec61-22* cells was monitored in liquid media either in UT condition or in presence of copper (0.6 mM). Graphs represent the absorbance at 600 nm values taken every 30′ for the indicated hours. DMSO, dimethyl sulfoxide; Tm, tunicamycin.

**Figure 7:**
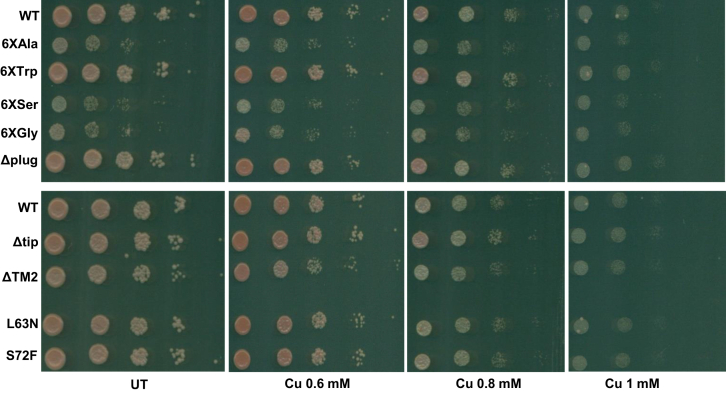
**Translocation-defective *prl* mutants do not show any resistancein the presence of copper.** Spot test assay of indicated WT and *prl*-positive mutants of sec61 (at 10-fold dilution, *left to right*) in plates containing increasing concentrations of copper CuCl_2_.2H_2_O (0.6, 0.8, and 1 mM). Untreated (UT) plate used as control shows uninhibited growth of WT and mutants. Cells were grown at 30 °C, and plates were scanned on fourth day. Representative images from three consistent independent repeats are shown.

Tm targets Alg7p to inhibit the N-linked glycosylation of proteins in yeast cells (48, 56). We have established that the Sec61p, an essential component of protein translocon channel, plays a role in mitigating copper toxicity. To establish the unique targeting of Sec61 by copper, we checked if the mutants are sensitive to either DTT or Tm. Our results (Fig. 6, *A*–*C*) show that reduced expression of Sec61 in the *DAmP* mutant does not lead to enhanced sensitivity in DTT or Tm treatment. Although the mutant shows sensitivity in DTT in the growth curve, there is no enhanced sensitivity toward Tm, which establishes that copper-exerted toxicity in mutant with reduced Sec61 level is selective. We also checked if treatment with Tm has any effect on the expression of *SEC61* levels. Fig. S2*B* shows that the levels of *SEC61* remain unaltered under Tm treatment. As previously stated, we found little difference in the *SEC61* expression of UT *versus* copper-treated samples. Thus, we surmised that, to inhibit the Sec61 protein, copper must be interacting with one or the other residues of the protein. To probe if there is a particular patch of residues in Sec61p, which mediates the toxicity of copper, we screened the well-established translocation-defective Sec61p strains (27) in copper and found that one of the mutants *sec61-22* is resistant to copper (Fig. 6, *D* and *E*). This mutant has four mutations in Sec61p polypeptide stretch L80M, V134I, M248V, L342S and has been shown to be defective in the preprocessing of CPY. Thus, it may happen that copper directly interacts with one or more of these residues to exert its toxicity while the mutation abrogates the interaction and so the mutant is resistant to the copper insult. Interestingly, one of the mutated residues (L80) lies right at the center of two residues, *viz.*, E79 and G81, which have been shown to exhibit *prl*-positive character when mutated as E79K and G81D, respectively. *prl* mutants of the Sec61 protein have been shown to be resistant to the decadepsipeptide compound, which blocks the entry of cotranslationally or post-translationally translocating substrates into the ER lumen. These mutants also allow the translocation of the defective signal sequence bearing substrates of the translocon. Thus, the mutants seem to destabilize the closed conformation of the channel, and so the sensitivity of cells resulting from blocking of the translocation of substrates by the compound is ameliorated (32). Similarly, a number of other *prl* mutants have been developed for the Sec61p mainly belonging to the plug and lateral gate domain region of the protein (57). If the L80M mutation also exhibits the *prl*-positive phenotype and is resistant to copper. Then, it may be concluded that copper binds to Sec61 when it is still closed or just before its opening so that the sec61 channel is blocked, which inhibits the translocation of the secretory proteins. To test the involvement of *prl* mutants in copper toxicity, we analyzed the growth of previously identified mutants, which were shown to be defective in translocation of post-translationally translocating proteins and showed *prl*-positive phenotype (32, 57, 58, 59). Figure 7 shows that none of the mutants showed resistance to different concentrations of copper treatment indicating that copper probably does not cause defect in translocation by stabilizing the closed confirmation of the sec61 channel. However, it is still possible that copper interacts with one or more selective residues of the sec61 protein to bring about a defect in the translocation of post-translationally translocating proteins.

## Discussion

**Figure 8:**
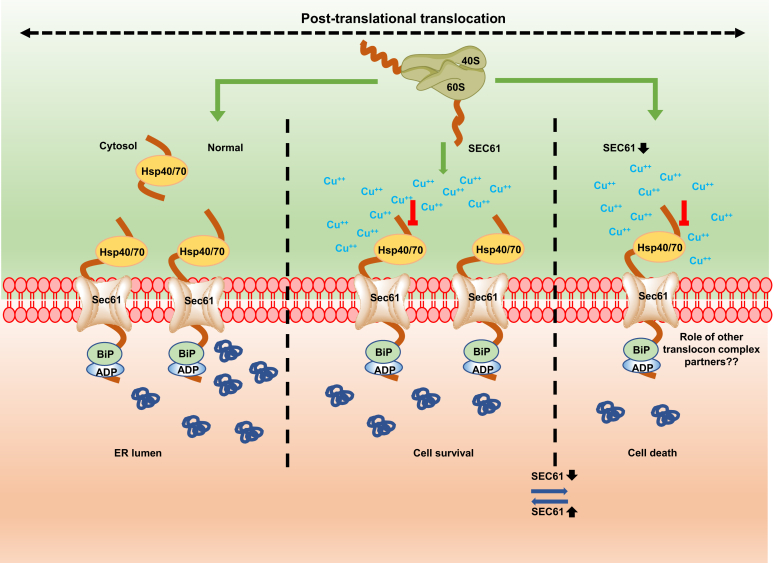
**Proposed model for copper-mediated accumulation of secretory proteins.** SRP-independent proteins utilize the Sec61 translocon complex to gain entry into the ER lumen. Copper targets Sec61 protein in yeast. In cells with already low levels of Sec61 protein, such an inhibition by copper almost blocks the entire function of translocating channel leading to lethality. Upon the restoration of Sec61 copy number, the lethality is rescued as some level of protein translocation is still possible. ER, endoplasmic reticulum; SRP, signal recognition particle.

Copper exerts toxic effects in the yeast system through multiple ways. Cells synthesize metallothionein and exporting proteins to curb the menace of free copper by sequestering and/or exporting it. Here, we show a new means through which copper exerts its toxic effect on the secretory proteins by targeting Sec61p, the principle translocon channel of the yeast system (Figure 8). We established that although treatment with Tm also leads to the accumulation of similar precursor deglycosylated Gas1, the effect is not mediated by targeting Sec61 as seen in the case of copper. Thus, copper uniquely targets the translocon to accumulate the precursor form of both Gas1p and CPY protein. Under normal conditions, the post-translational translocation is mediated by the trimeric complex in association with the partners Sec62 and Sec63. In the presence of copper, however, the process is most probably inhibited because of the interaction of copper with either one or more L80, V134, M248, and L342 residues of Sec61. In addition, when cells with low copies of Sec61 are exposed to the metal, such an inhibition becomes lethal. However, when the copies of Sec61 are restored in such cells, the lethality is ameliorated (Fig. S3, *A*–*D*) (Fig. 8). As stated earlier, Sec61 translocon comprises of Sec61, Sbh1, and Sss1, all of which together form the protein-conducting channel. Sbh1p interacts with Sec61 and Sss1p through its TM domain and contributes to the functioning of the translocon (16). Stability of the translocon complex would be challenged because of lower levels of Sec61 in *sec61-DAmP* strains. Thus, it would be interesting to find out the effect of copper on Sbh1, Sec71, Sec72, deletion or *sec62-DAmP*, *sec63-DAmP* in the *sec61*-*DAmP* background (Fig. 8). Copper has been shown to play a role in controlling the glycosylation levels of the secretory proteins (33). However, no mechanism has been proposed for the observation. Our study provides a clue that probably the targeting of the translocon reduces the glycosylation of these proteins. Studies have reported that the protein Gas1p accumulates in its precursor form in Tm-treated condition. *sec61-DAmP* strains are defective in translocation of secretory proteins and are sensitive to copper. Furthermore, *sec61-DAmP* strain accumulates the precursor forms of Gas1p and CPY (preproCPY) (27, 40). We obtained similar results upon treating the yeast cells with copper. Copper toxicity of s*ec61-DAmP* strains was essentially reversed when Sec61 levels were restored using the *SEC61*-expressing plasmid. Thus, copper treatment leads to throttling of precursor Gas1p and CPY by affecting the translocation through the Sec61 channel (Fig. 8). Interestingly, GPI-anchored proteins are involved in many crucial functions within the cells as well as the proteins of many parasites (60, 61, 62). *Trypanosoma brucei* causing African sleeping sickness is one such protozoan, which uses variant surface glycoprotein (VSG) as a means to evade the host immune response (63). Its success in the evasion process is due to extreme variation of the VSGs, which become difficult to target (64). However, one common feature of all the VSGs is that they are all GPI anchored. Nascent form of VSG encodes a hydrophobic signal sequence at the N-terminal region, which targets the protein toward the ER lumen through the Sec61 translocon pore. Since copper targets the Sec61 protein in yeast and Sec61 is highly conserved across different species (14, 20), our results can pave way for copper-containing specific drugs targeting the Sec61 of *T. brucei*, which can help in reducing the menace of the parasite by reducing the VSG. The quadruple mutant *sec61-22* bearing mutations at L80M, V134I, M248V, and L342S was resistant to copper. On mapping their positions, L80, V134, and M248 were found to be part of the membrane and belonging to TM2, TM3 and TM6, respectively, and L342 was shown to be part of the loop between TM 7 and TM 8 (L7/8) (13, 65). Recent cryo-EM technique with high-resolution data on the structure of post-translational translocation from *S. cerevisiae* has revealed that TMs of Sec63 located at the back of Sec61 channel interact with the protein *via* TM1 and TM5 (66). It is to be noted that the hinge movement between two halves of the Sec61 (TM1–5 and TM6–10) plays an important role in opening of the channel pore (67). The contact between Sec62 and Sec61 channel also occurs *via* TM1 of Sec62 with TM3 of Sec61 protein interaction. It is also important to note that the plug position is maintained through the motion of TM7–8 of Sec61p. Extent of lateral gate opening in the Sec61 translocon is guided by the disengagement of sec62-TM2. This movement is mediated by the rotation of TM7, TM8, and also the loop in between these two TMs (L7/8) of Sec61, making it important for the overall functioning of translocon (67). Given the involvement of these Sec61 TMs in the interaction with its partners, Sec62 and Sec63, it is tempting to speculate that copper may involve in disrupting the interactions important for the stability of the complex. Thus, perturbation of complex stability may hinder the translocation of post-translationally translocating proteins.

One of the major ways through which copper induces cytotoxicity is by participating in Fenton-like reaction leading to ROS generation (34). Copper also directly binds to glutathione, thereby reducing its levels inside the cells. With reduced glutathione levels, the effect of ROS becomes even more pronounced. Hydroxy radicals/free radicals generated as a result of these reactions are powerful ROS, which can have deleterious effects on the function or structure of diverse biomolecules, such as lipids, DNA, and proteins (35). Increasing evidences suggest that such free radicals can cause fragmentation or conformational change of proteins through modification of their amino acid or involve in lipid peroxidation. Tyrosine, tryptophan, phenylalanine, praline, and histidine residues in proteins may get oxidized as a result of ROS (68). Thus, the affected protein may lose its function. In addition, lipid peroxidation may also damage the proteins in the vicinity through protein and lipid aldehyde crosslinkage (69). The function of Sec61p, a TM protein, may get adversely affected by copper-induced lipid peroxidation or ROS. Interestingly, GSH is imported into ER by facilitated diffusion through the Sec61 channel (70). Since copper directly binds to GSH, its concentration inside the ER may increase leading to selective increase in ER ROS. Also, copper may interact with Sec61 to inhibit its function when the GSH–copper complex is passing through it.

Sec61 forms the common component for both the cotranslational and post-translational protein translocation complex (71). Here, we have shown that the translocation of post-translationally targeting proteins is getting affected in the presence of copper. Thus, it would be fascinating to study the translocation of the cotranslationally targeting proteins like DPAPB in the presence of copper (45). Previous studies have elucidated the role of complex molecules in blocking the translocation of the substrates (32). To our knowledge, this is the first time that copper ions have been shown to be able to affect the translocation process.

## Experimental procedures

### Yeast strains and growth conditions

Unless stated otherwise, strains of *S. cerevisiae* S288C were from background of BY4743. Cells were grown in synthetic complete (SC) (0.18 g all amino acid mix with nutrients, 0.17 g yeast nitrogen base without ammonium sulphate [HiMedia], 0.5 g ammonium sulphate, and 2 g glucose with 2 g agar [solid medium] or without it [liquid medium] in 100 ml water according to standard protocol) or SC-Leu/SC-His media at 30 °C under optimal conditions. Complete list of primers, strains, and plasmids is available in Tables S1–S3. *sec61-DAmP* and *sec62-DAmP* strains were confirmed using the following confirmatory primers (Table S1) (53).

### Growth assays

#### Spot test assays

Overnight grown saturated yeast cultures were taken in equal number (absorbance at 600 nm = 1) and serially diluted by 10-fold three or four times. Equal numbers of cells were individually spotted on UT and treated plates. Dried plates were incubated for 2 to 4 days at optimal temperature of 30 °C, and data were recorded through HP ScanJet (G2410).

#### Growth curve assays

The effect of different stress-causing agents on growth of yeast cells was visualized through growth curve assay. Briefly, saturated overnight grown yeast culture was secondary inoculated, and equal number of cells (absorbance at 600 nm = 0.1/0.2) was seeded in 96-well plate (Nunc) under UT/treated conditions. Growth was recorded by acquiring absorbance at 600 nm every 30′ for 2 days in automated BioTek plate reader.

### Protein extraction

Whole-cell extracts from yeast cells were prepared through the trichloroacetic acid (TCA) method (72) with few modifications. Briefly, the yeast cells harvested at different conditions were washed with 20% TCA and stored at −80 °C. At the time of extraction, cells were resuspended in 20% TCA, and equal volume of glass beads was added followed by vigorous vortexing to lyse the cells. The precipitated protein extract was centrifuged at 7000 rpm for 10′ at 4 °C. The supernatant was discarded, and the pellet was washed with 0.5 M Tris–Cl (pH 7.5). The sample was resuspended in 1× loading buffer and heated at 100 °C for 10′, following which it was centrifuged at maximum rpm for 15′ to separate the insoluble debris, and the supernatant containing the protein was used for immunoblotting.

### Immunoblotting

SDS-PAGE was performed to resolve the proteins. Resolved proteins were transferred to nitrocellulose membrane in transfer buffer containing Tris–Cl (pH 7.5), glycine, methanol, and SDS for 90′ at 4 °C using mini wet transfer apparatus from Bio-Rad. After transfer, the membrane was incubated in 2.5% bovine serum albumin (catalog no.: MB083; HiMedia) for 45′ at room temperature for blocking. The membrane was incubated in primary antibody for 90′ at room temperature, following which it was washed with 1× Tris-buffered saline with Tween-20 (containing Tris–Cl [pH 7.5], NaCl, and Tween-20) to remove nonspecific binding of the primary antibody. Washed membrane was then incubated in IR dye–labeled secondary antibody for 45′ at room temperature, following which it was washed with 1× Tris-buffered saline with Tween-20 to remove nonspecific binding of the secondary antibody. Membrane was visualized using LI-COR infrared imaging system. Primary antibodies used for immunoblotting were as follows: α-GFP (catalog no.: G1544; Sigma), α-TBP1 polyclonal antiserum from rabbit (generated in laboratory), α-Myc monoclonal antibody (catalog no.: MA1-980; Invitrogen), α-Sec61 (kind gift from R. Schekman), goat anti-rabbit IgG secondary antibody (catalog no.: A32734; Invitrogen), and goat antimouse IgG secondary antibody (catalog no.: 926-32210; Odyssey).

The percentage of Gas1 or CPY immature form was calculated by normalizing the intensity of mature band and that of immature band. The normalized intensities were added to get the total Gas1 or CPY protein level. Percent of immature protein w.r.t. total protein level was calculated as (normalized intensity of immature protein/intensity of total protein) ∗ 100.

### Transformations and tagging

Template for tagging CPY at the C terminus was generated through two-step PCR of pFA6a-13myc plasmid and the primers specific for CPY C-terminal tagging (73). The purified product was used as template for the transformation of yeast cells (74), and the positive colonies were selected using SC-His plates. The tagging was confirmed by the Western blot of the cells using the α-Myc antibody.

### DNA isolation from yeast

Cells were harvested from overnight culture, and DNA was extracted (75). Briefly, cells were washed with ice-cold water and centrifuged at 4000 rpm for 5′ at 4 °C. The pellet was mixed with 300 μl 0.2% SDS, vortexed, and incubated at 100 °C for 15′, following which NaCl was added to a concentration of 100 mM and the mixture was vortexed and centrifuged at 10,000 rpm for 15′ at 4 °C. The supernatant was transferred to a new vial and incubated with RNase-A (100 μg/ml; catalog no.: 10109169001; Sigma) at 37 °C for 15’. The resulting lysate was added with equal volume of phenol (pH 8):chloroform:isoamylalcohol (25:24:1), vortexed, and centrifuged at 14,000 rpm for 15′ at room temperature. The aqueous phase containing DNA was taken to a new tube and precipitated with absolute ethanol at −80 °C for 45’. DNA obtained postcentrifugation was air dried for 30′ and resuspended in Milli-Q and transferred to 4 °C.

Semiquantitative PCR was performed as per the manufacturer’s instructions (KAPA Taq DNA Polymerase; catalog no.: KK1015) to check the levels of different genes.

### RNA extraction and quantitative real-time PCR of genes

Primary cultures of strains were used for secondary culture and grown till an absorbance of 0.8 to 1 at 600 nm was reached. The cells were harvested, and RNA was extracted using the hot phenol method (76). Normalized RNA was used to synthesize the complementary DNA by iScript cDNA synthesis kit (catalog no.: 1708891; Bio-Rad). quantitative RT–PCRs of respective genes were performed with ABI-7300 RT–PCR with Sequence Detection System v1.4 (Applied Biosystems) according to the instructions by manufacturer using TB Green Premix Ex Taq II (catalog no.: RR820B; Takara). Relative mRNA expression was calculated according to the 2^−ΔΔCT^ method (77). The relative expression fold changes were calculated using the *ACT1* gene as control. Data are represented as mean ± SD of three biological replicates.

### EndoH glycosidase assay

Proteins for EndoH assay were extracted as previously described (52) with some modifications. Briefly, approximately 30 cells were resuspended at an absorbance at 600 nm in Tris–HCl (50 mM, pH 7.5) buffer containing 1 mM PMSF and broken with vortexing in the presence of glass beads. The resulting lysate was clarified by centrifugation at 5000 rpm for 5 min at 4 °C. The supernatant was transferred to a fresh tube and denatured using the denaturation buffer (40 mM DTT and 0.5% SDS) and incubation for 10 min at 100 °C. Following the denaturation, the samples were diluted twofold with final concentration of SDS at 0.25% and sodium acetate at 50 mM, pH 6. Finally, the sample was divided into two sections: one was treated with endoglycosidase H (catalog no.: P0702S; New England Biolabs), whereas the other was only incubated with buffer for 2 h at 37 °C. Postincubation, the samples were mixed with Laemmli buffer, resolved in SDS-PAGE, and immunoblotted.

### Fluorescence microscopy

Gas1-GFP transformed GSHY583 yeast cells grown overnight in Sc-Leu medium were diluted to an absorbance of 0.2 at 600 nm and allowed to reach the exponential phase of absorbance of ∼1 at 600 nm. At this phase, the cells were treated with Cu at 1 mM or UT and grown for 90 min. Postincubation, the cells were harvested and washed twice with 1× PBS. Images were acquired using ZEISS-Apotome.2 fluorescence microscope (ZEISS). The λ excitation/λ emission used for GFP is 450 to 490 nm/500 to 550 nm and for dsRed is 538 to 562 nm/570 to 640 nm. Processing of the image was done using ZEN 2012 software (ZEISS).

## Data availability

All the relevant data are contained within the article and the supporting information.

## Supporting information

This article contains supporting information (27, 32, 38, 40, 41, 44, 53).

## Conflict of interest

The authors declare that they have no conflicts of interest with the contents of this article.

## Supporting information

revised supporting information
