# Supplementary material for: Copper inhibits protein maturation in the secretory pathway by targeting the Sec61 translocon in Saccharomyces cerevisiae
Source: J Biol Chem. 2022 Jun 20;298(8):102170. doi: 10.1016/j.jbc.2022.102170 (PMC9304788; doi:10.1016/j.jbc.2022.102170)
Supplement: revised supporting information [file mmc1.docx]

**Supporting information**

**Copper inhibits protein maturation in the secretory pathway by targeting the Sec61 translocon in *Saccharomyces cerevisiae***

Nitu Saha^1^, Raghuvir Singh Tomar^1†^

^1^Laboratory of Chromatin Biology, Department of Biological Sciences, Indian Institute of Science Education and Research Bhopal, 462066, Madhya Pradesh, India.

^†^To whom correspondence may be addressed:

Email: rst@iiserb.ac.in

**Figure S1. Dose response of Gas1-GFP transformed and CPY-myc tagged cells towards copper.** *A*, growth of Gas1-GFP transformed BY4743 (WT) cells was monitored in different concentrations of copper (indicated) *B*, growth of CPY-myc tagged BY4741 (WT) cells was monitored in different concentrations of copper (indicated). Both the growth curves show measurement of absorbance at 600nm values taken every 30’ for the indicated hours.

**Figure S2.** ***SEC61* expression shows subtle change in copper treated cells.** *A* and *B*, *SEC61* and *SEC66* level was examined in cDNA prepared from WT (G418) and s*ec61-DAmP* treated cells with indicated concentrations of copper or Tm for the indicated time. *ACT1* expression acts as the control band.

**Figure S3. *sec61-DAmP* strain sensitivity towards copper is ameliorated upon restoration of SEC61 levels.** *A*, spot-test assay of wild-type BY4741 with G418 in HO locus [WT (G418)], *sec61-DAmP* transformed with *EV* or *SEC61* expression plasmid (at 10-fold dilution left to right) to test sensitivity in plates containing increasing concentration of copper CuCl_2_·2H_2_O (1 mM, 1.25 mM, 1.5 mM). Untreated (UT) plate used as control shows uninhibited growth. Cells were grown at 30 °C and plates were scanned on 4^th^ day. Representative images from three consistent independent repeats are shown. *B*, growth of the same transformed cells was monitored in liquid media either in untreated (UT) condition or in presence of copper (1 mM). Graphs represent the absorbance at 600 nm values taken every 30’ for the indicated hours. *C*, *SEC61* mRNA level was examined in same cells and the expression of *ACT1* was used as control. *D*, statistical analysis of the semiquantitative PCR from three independent biological repeats was performed using unpaired Student’s *t*-test with Welch’s correction (in graphpad prism 8, GraphPad, San Diego, CA, USA). ***P* ≤ 0.01, ****P* ≤ 0.001. *E*, immunoblots showing the accumulation of preproCPY in *sec61-DAmP* and rescue of immature protein by *SEC61* expression plasmid. Indicated strains were grown till an absorbance of ~1 at 600 nm and harvested. Proteins extracted from these cells were subjected to western blot (described in Experimental Procedures section). TBP and H3 levels served as control. *F*, quantification of the western blots (w.r.t. TBP) from (n=3) independent biological repeats (quantification described in Experimental Procedures). Graphs plotted as mean ± SD.

**Table S1: List of primers used in this study**

| **Primers Name** | **Primers Sequence (5′-3′)** | **References** |
| --- | --- | --- |
| ***SEC61*-F** | **TTCTGGAGAGCGTTTGCTCC** | **This study** |
| ***SEC61*-R** | **AAAGAGGAAGATGGCCACGG** | **This study** |
| ***sec61-DAmP* Confirmatory-F** | **CCCAATCATGTTGCAGAGTG** | **(1)** |
| ***sec61-DAmP* Confirmatory-R** | **CTGCAGCGAGGAGCCGTAAT** | **(1)** |
| ***sec62-DAmP* Confirmatory-F** | **GATTGAAGCCAAGCAAGGAC** | **(1)** |
| ***sec62-DAmP* Confirmatory-R** | **CTGCAGCGAGGAGCCGTAAT** | **(1)** |
| ***SEC72*-F** | **CGCCTTGGGAAGCTTTTGC** | **This study** |
| ***SEC72*-R** | **AGCCTCTTCCCACTGTCTCA** | **This study** |
| ***CPY* myc-F** | **AAGTATGGTTAACGAATGGATCCACGGTGGTTTCTCCTTACGGATCCCCGGGTTAATTAA** | **This study** |
| ***CPY* myc-R** | **GTAGCTGATAATAAAAACGGTATGCCTACACATACACGCTGAATTCGAGCTCGTTTAAAC** | **This study** |
| ***SEC66*-F** | **GGCCGCTTTATTGAACAGAGGA** | **This study** |
| ***SEC66*-R** | **GCCCGGTTGCAATCTTTCAG** | **This study** |
| ***ACT1*-F** | **TCGTCGGTAGACCAAGACAC** | **This study** |
| ***ACT1*-R** | **TTCTTCTGGGGCAACTCTCA** | **This study** |

**Table S2: List of strains used in this study**

| **Strain Name** | **Genotype** | **Reference** |
| --- | --- | --- |
| **BY4743** | *MATa/α his3Δ1/his3Δ1 leu2Δ0/leu2Δ0 LYS2/lys2Δ0 met15Δ0/MET15 ura3Δ0/ura3Δ0* | Yeast Knockout Collection Open Biosystems (YKO-OB) |
| **BY4741** | ***MATa his3Δ1 leu2Δ0 met15Δ0 ura3Δ0*** | Yeast Knockout Collection Open Biosystems (YKO-OB) |
| **BY4741with G418 in HO Locus (WT for DamP strains and Sec72 Δ)** | ***his3∆1 leu2∆0 met15∆0 ura3∆0; HO:G418*** | **(2)** |
| ***sec61-DAmP*** | ***his3∆1 leu2∆0 met15∆0 ura3∆0 sec61-DAmP::KanR*** | **(2)** |
| ***sec62-DAmP*** | ***his3∆1 leu2∆0 met15∆0 ura3∆0 sec62-DAmP::KanR*** | **(2)** |
| ***sec72Δ*** | ***his3∆1 leu2∆0 met15∆0 ura3∆0 ∆sec72::KanR*** | **(2)** |
| **BY4741 (WT)** | ***MATa his3∆1 leu2∆0 met15∆0 ura3∆0*** | **(2)** |
| **RSY1293** | ***mat*a*, can1-100, leu2-3,112, his3-11,15, trp1-1, ura3-1,ade2-1, sec61*<*HIS3* (pDQ1 [sec61-*his6*])** | **(3)** |
| **RSY1294** | **same as RSY1293 but sec61-32** | **(3)** |
| **RSY1296** | **same as RSY1293 but sec61-86** | **(3)** |
| **RSY1297** | **same as RSY1293 but sec61-7** | **(3)** |
| **RSY1299** | **same as RSY1293 but sec61-10** | **(3)** |
| **RSY1300** | **same as RSY1293 but sec61-11** | **(3)** |
| **RSY1302** | **same as RSY1293 but sec61-22** | **(3)** |
| **RSY1303** | **same as RSY1293 but sec61-23** | **(3)** |
| **RSY1304** | **same as RSY1293 but sec61-24** | **(3)** |
| **RSY1428** | **same as RSY1293 but SEC61 (pSEC61-wt)** | **(3)** |
| **RSY1429** | **same as RSY1293 but sec61-110** | **(3)** |
| **WCG4a** | **WCG4a** | **(3)** |
| **GSHY583** | **BY4742 ss-dsRed-HDEL::natNT1** | **(4)** |
| **61wt** | **mat alpha, ura3-1, leu2-3,-112, his3-11,-15, trp1-1, ade2-1, can1-100, sec61::HIS3(H6-sec61-YCplac111)** | **(5)** |
| **6xAla** | **mat alpha, ura3-1, leu2-3,-112, his3-11,-15, trp1-1, ade2-1, can1-100, sec61::HIS3(H6-sec61-82/I86/181/185/294/450 A- YCplac111)** | **(5)** |
| **6xSer** | **mat alpha, ura3-1, leu2-3,-112, his3-11,-15, trp1-1, ade2-1, can1-100, sec61::HIS3 (H6-sec61-82/I86/181/185/294/450 S- YCplac111)** | **(5)** |
| **6xGly** | **mat alpha, ura3-1, leu2-3,-112, his3-11,-15, trp1-1, ade2-1, can1-100, sec61::HIS3 (H6-sec61-82/I86/181/185/294/450 G- YCplac111)** | **(5)** |
| **6xTrp** | **mat alpha, ura3-1, leu2-3,-112, his3-11,-15, trp1-1, ade2-1, can1-100, sec61::HIS3 (H6-sec61-82/I86/181/185/294/450 W- YCplac111)** | **(5)** |
| **∆2a** | **mat alpha, ura3-1, leu2-3,-112, his3-11,-15, trp1-1, ade2-1, can1-100, sec61::HIS3 (H6-sec61-∆plug-YCplac111)** | **(5)** |
| **∆tip** | **mat alpha, ura3-1, leu2-3,-112, his3-11,-15, trp1-1, ade2-1, can1-100, sec61::HIS3 (H6-sec61-∆tip-YCplac111)** | **(5)** |
| **∆2b** | **mat alpha, ura3-1, leu2-3,-112, his3-11,-15, trp1-1, ade2-1, can1-100, sec61::HIS3 (H6-sec61-∆2b-YCplac111)** | **(5)** |
| **L63N** | **mat alpha, ura3-1, leu2-3,-112, his3-11,-15, trp1-1, ade2-1, can1-100, sec61::HIS3 (H6-sec61-L63N-YCplac111)** | **(5)** |
| **S72F** | **mat alpha, ura3-1, leu2-3,-112, his3-11,-15, trp1-1, ade2-1, can1-100, sec61::HIS3 (H6-sec61-S72F-YCplac111)** | **(5)** |

**Table S3: List of plasmids used in the study**

| **Plasmid Name** | **References** |
| --- | --- |
| **pRS415 (EV for Gas1-GFP)** | **(6)** |
| **Gas1-GFP pRS415** | **(6)** |
| **RSB 205 (EV for *SEC61*)** | **(3,7)** |
| **RSB 517 (*SEC61* expressed at the WT levels)** | **(3,7)** |

**References**

1. Breslow, D. K., Cameron, D. M., Collins, S. R., Schuldiner, M., Stewart-Ornstein, J., Newman, H. W., Braun, S., Madhani, H. D., Krogan, N. J., and Weissman, J. S. (2008) A comprehensive strategy enabling high-resolution functional analysis of the yeast genome. *Nature methods* **5**, 711-718

2. Ast, T., Cohen, G., and Schuldiner, M. (2013) A network of cytosolic factors targets SRP-independent proteins to the endoplasmic reticulum. *Cell* **152**, 1134-1145

3. Pilon, M., Romisch, K., Quach, D., and Schekman, R. (1998) Sec61p serves multiple roles in secretory precursor binding and translocation into the endoplasmic reticulum membrane. *Molecular biology of the cell* **9**, 3455-3473

4. Suresh, H. G., da Silveira Dos Santos, A. X., Kukulski, W., Tyedmers, J., Riezman, H., Bukau, B., and Mogk, A. (2015) Prolonged starvation drives reversible sequestration of lipid biosynthetic enzymes and organelle reorganization in Saccharomyces cerevisiae. *Molecular biology of the cell* **26**, 1601-1615

5. Junne, T., Wong, J., Studer, C., Aust, T., Bauer, B. W., Beibel, M., Bhullar, B., Bruccoleri, R., Eichenberger, J., Estoppey, D., Hartmann, N., Knapp, B., Krastel, P., Melin, N., Oakeley, E. J., Oberer, L., Riedl, R., Roma, G., Schuierer, S., Petersen, F., Tallarico, J. A., Rapoport, T. A., Spiess, M., and Hoepfner, D. (2015) Decatransin, a new natural product inhibiting protein translocation at the Sec61/SecYEG translocon. *J Cell Sci* **128**, 1217-1229

6. Ha, C. W., Kim, K., Chang, Y. J., Kim, B., and Huh, W. K. (2014) The beta-1,3-glucanosyltransferase Gas1 regulates Sir2-mediated rDNA stability in Saccharomyces cerevisiae. *Nucleic acids research* **42**, 8486-8499

7. Pilon, M., Schekman, R., and Romisch, K. (1997) Sec61p mediates export of a misfolded secretory protein from the endoplasmic reticulum to the cytosol for degradation. *The EMBO journal* **16**, 4540-4548
